# The impact of ultra-high-density mapping on long-term outcome after catheter ablation of ventricular tachycardia

**DOI:** 10.1038/s41598-022-12918-7

**Published:** 2022-06-01

**Authors:** Ruben Schleberger, Jana M. Schwarzl, Julia Moser, Moritz Nies, Alexandra Höller, Paula Münkler, Leon Dinshaw, Christiane Jungen, Marc D. Lemoine, Philippe Maury, Frederic Sacher, Claire A. Martin, Tom Wong, Heidi L. Estner, Pierre Jaïs, Stephan Willems, Christian Eickholt, Christian Meyer

**Affiliations:** 1 Department of Cardiology, University Heart and Vascular Center Hamburg, University Medical Center Hamburg-Eppendorf, Hamburg, Germany; 2 Center of Experimental Medicine, Institute of Medical Biometry and Epidemiology, University Medical Center Hamburg-Eppendorf, Hamburg, Germany; 3 DZHK (German Center for Cardiovascular Research), Partner Site Hamburg/Kiel/Lübeck, Berlin, Germany; 4 Department of Cardiology, Willem Einthoven Center for Cardiac Arrhythmia Research and Management, Leiden University Medical Center, Leiden, The Netherlands; 5 Department of Cardiology, University Hospital Rangueil, Toulouse, France; 6 LIRYC Institute, CHU Bordeaux, University of Bordeaux, Bordeaux, France; 7 Royal Papworth Hospital, National Health Service Foundation Trust, Cambridge, UK; 8 Heart Rhythm Center, The Royal Brompton and Harefield NHS Foundation Trust, Imperial College London, London, UK; 9 Department of Internal Medicine I - Cardiology, University Hospital Munich, Ludwig-Maximilian University Munich, Munich, Germany; 10 Department of Cardiology, Asklepios Hospital St. Georg, Hamburg, Germany; 11 Department of Cardiology, Cardiac Neuro- and Electrophysiology Research Consortium (cNEP), EVK Düsseldorf, Düsseldorf, Germany; 12 Cardiac Neuro- and Electrophysiology Research Consortium (cNEP), Medical Faculty, Heinrich Heine University Düsseldorf, Düsseldorf, Germany

**Keywords:** Cardiology, Interventional cardiology

## Abstract

Ultra-high-density (UHD) mapping can improve scar area detection and fast activation mapping in patients undergoing catheter ablation of ventricular tachycardia (VT). The aim of the present study was to compare the outcome after VT ablation guided by UHD and conventional point-by-point 3D-mapping. The acute and long-term ablation outcome of 61 consecutive patients with UHD mapping (64-electrode mini-basket catheter) was compared to 61 consecutive patients with conventional point-by-point 3D-mapping using a 3.5 mm tip catheter. Patients, whose ablation was guided by UHD mapping had an improved 24-months outcome in comparison to patients with conventional mapping (cumulative incidence estimate of the combination of recurrence or disease-related death of 52.4% (95% confidence interval (CI) [36.9–65.7]; recurrence: n = 25; disease-related death: n = 4) versus 69.6% (95% CI [55.9–79.8]); recurrence: n = 31; disease-related death n = 11). In a cause-specific Cox proportional hazards model, UHD mapping (hazard ratio (HR) 0.623; 95% CI [0.390–0.995]; *P* = 0.048) and left ventricular ejection fraction > 30% (HR 0.485; 95% CI [0.290–0.813]; *P* = 0.006) were independently associated with lower rates of recurrence or disease-related death. Other procedural parameters were similar in both groups. In conclusion, UHD mapping during VT ablation was associated with fewer VT recurrences or disease-related deaths during long-term follow-up in comparison to conventional point-by-point mapping. Complication rates and other procedural parameters were similar in both groups.

## Introduction

Episodes of ventricular tachycardia (VT) and shocks by implanted cardioverter-defibrillators (ICD) are associated with an increased mortality and morbidity in patients with structural heart disease^1^. Besides optimal treatment of heart failure and concomitant conditions^2,3^, catheter ablation has become the gold standard to prevent recurring episodes^4^. Still, recurrences are sometimes seen, especially in patients with advanced stages of structural heart disease and heart failure^5^. Often a substrate-based ablation approach, targeting low-voltage areas and local abnormal ventricular activation (LAVA) is chosen, especially when mapping during active VT is hemodynamically not tolerated^6^. Recent studies have shown that ablation covering the entire substrate achieves a better outcome than incomplete substrate modification^7^. Yet, the detection of abnormal signals may depend on the hard- and software used, as subtle non-transmural myocardial scars might not always be identified with 3.5 mm tip catheters^8^. Multi-electrode mapping of abnormal electrograms has been found to increase the sensitivity of identification of scar areas^7^. Small and closely spaced electrodes facilitate the depiction of distinct electrograms as e.g., diastolic signals and might improve the differentiation between near- and far-field signals^9^.

It remains to be demonstrated, whether these insights into the mechanisms of arrhythmia are only of academic interest or if they improve ablation success. Therefore, we aimed to investigate if ablation guided by ultra-high-density (UHD) mapping leads to a better ablation outcome than conventional point-by-point 3D-mapping.

## Methods

### Study design

**Figure 1:**
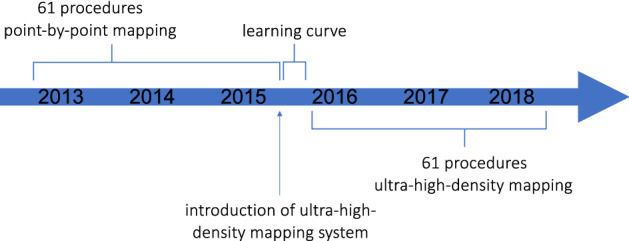
Single center sequential comparison study. The figure depicts the design of the present study. The acute and long-term outcome of patients whose ablation of ventricular tachycardia (VT) was guided by ultra-high-density mapping (UHD group) was compared to patients with conventional point-by-point 3D-mapping (conventional mapping group). Ultra-high-density mapping was established at the study center in 2015. Sixty-one consecutive patients with UHD mapping were included into the study. Patient selection started after a six-months run-in period after introduction of the system to avoid a learning-curve-effect. The patients’ outcome was compared to 61 consecutive patients that had a VT ablation guided by conventional 3D-mapping between 2013 and 2015, before introduction of UHD mapping.

This single center sequential comparison included 122 patients that presented for ablation of scar-related VT at our tertiary care hospital (study design see Fig. 1). The acute and long-term outcome of patients with UHD mapping was retrospectively compared to patients with conventional point-by-point 3D-mapping. Sixty-one consecutive patients (2016–2018) had ablation guided by UHD mapping (UHD group; 64-electrode mini-basket catheter in conjunction with a 3.5 mm tip ablation catheter). UHD mapping was introduced at the study center in 2015; patient inclusion started six months after introduction to minimize a learning curve effect. The patient data was compared to 61 consecutive patients with conventional point-by-point 3D-mapping (conventional mapping group; 3.5 mm tip catheter only), treated before the introduction of UHD mapping (2013–2015). Patients with idiopathic VT were excluded from the study.

### Electrophysiological evaluation and instrumentation

Conscious sedation was administered to most patients except for a few that required general anaesthesia. Reversible causes of VT were excluded before the procedure. Detailed procedural methods have been described before^10^. Briefly, the catheter setting consisted of a 6 French (F) quadripolar diagnostic catheter, placed in the right ventricular (RV) apex to induce VT by programmed stimulation with a fixed protocol. A catheter in the coronary sinus served as a reference for the 3D-electroanatomical mapping system in most cases. Unfractionated Heparin was administered intravenously to maintain an activated clotting time > 300 s in patients with UHD mapping and > 250 s in patients with conventional mapping during the procedure.

### Ultra-high-density 3D-mapping

UHD mapping was performed using the Rhythmia™ (Boston Scientific, Marlborough, MA, USA) mapping system as previously described^10,11^. An expandable, 64-polar mini-basket catheter (Orion™, Boston Scientific) comprising eight splines with eight electrodes each (electrode spacing 2.5 mm, electrode surface area 0.4 mm^2^) and an open-irrigated 3.5 mm tip mapping and ablation catheter (INTELLANAV™ OI / IntellaNAV MIFI™ OI, Boston Scientific) were introduced into the left ventricle (LV)^12^. Access to the LV was gained either by transseptal access after a single transseptal puncture using a fixed curve long sheath (SL0, 8.5 F, St. Jude Medical, Saint Paul, MN, USA; for ablation catheter) and a long steerable sheath (Agilis large curve, 8.5 F, St. Jude Medical, for mini-basket catheter) or by retrograde aortic access (Terumo 8 F, Leuven, Belgium).

**Figure 2:**
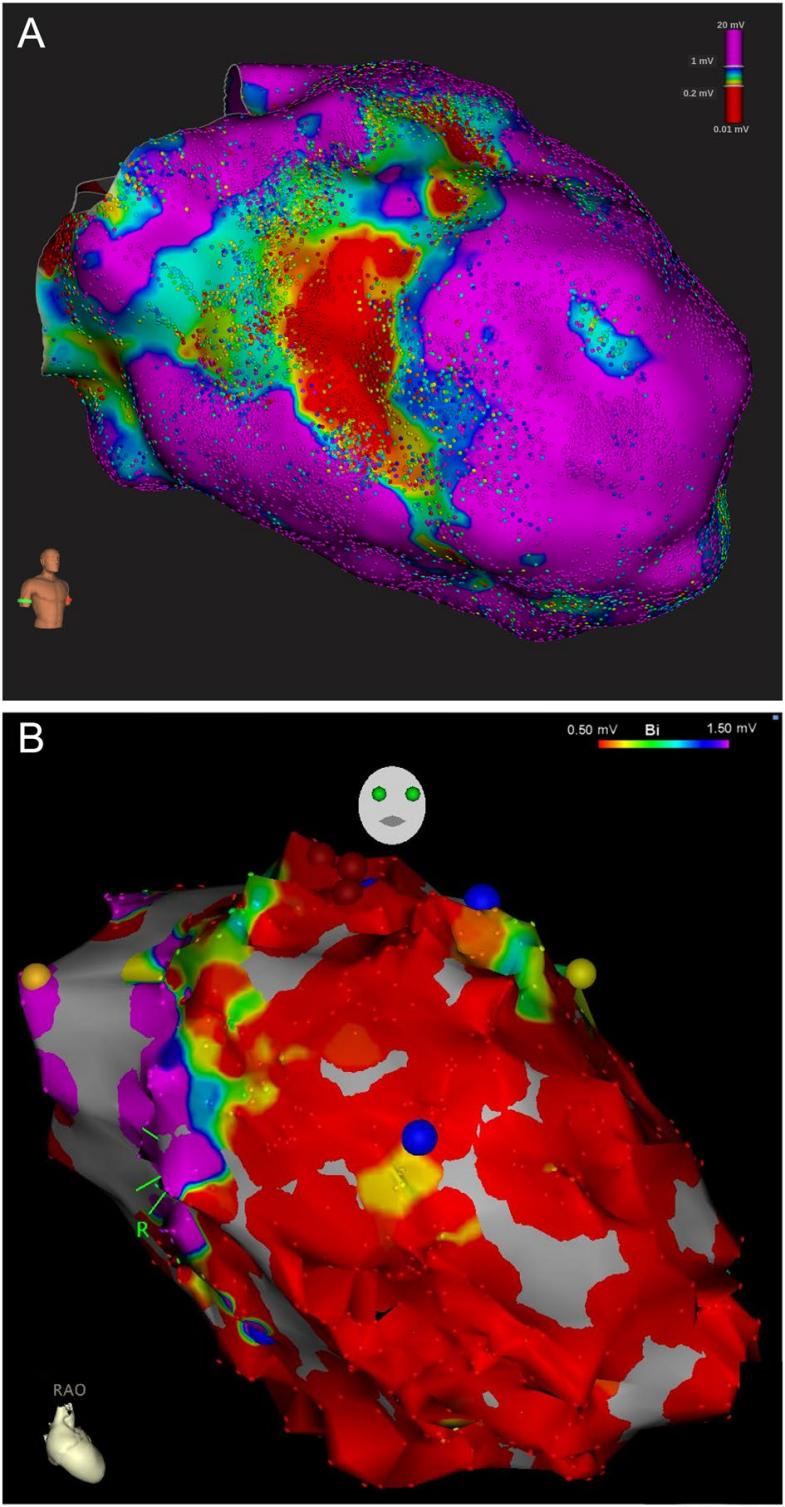
Substrate maps of the left ventricle. (**A**) Ultra-high-density map. (**A**) shows an ultra-high-density map of the left ventricle, created with a basket catheter (34,692 points). (**B**) Conventional point-by-point map. (**B**) shows a conventional map of the left ventricle, created by point-by-point mapping with a single tip catheter (889 points). Both maps are shown in the projection right anterior oblique. The colour scale symbolizes the myocardial voltage, the range is displayed in the right upper corner. The large blue and yellow balls in **B** mark abnormal electrograms. Bi indicates bipolar; mV, millivolt; RAO, right anterior oblique.

At first, an electroanatomical substrate map was acquired with the basket catheter and subsequently completed with the single-tip ablation catheter in less accessible areas (see Fig. 2A). Following this, if hemodynamically tolerated, VT was induced and activation mapping during VT was performed. In patients presenting with VT at the beginning of the procedure, activation mapping was conducted before substrate mapping. Electrogram annotation was performed automatically by the mapping system (Lumipoint™ software module) as previously described^10^.

In line with previous studies for scar demarcation a bipolar endocardial voltage of 0.2/1 mV (dense scar < 0.2 mV, border zone 0.2–1.0 mV, healthy tissue > 1.0 mV) with individual adaptation was chosen^10,13^. All voltage maps were generated during sinus rhythm or paced rhythm from coronary sinus or ventricle. Whenever VT was inducible and hemodynamically tolerated, activation mapping was performed. Hemodynamic instability was defined as mean arterial blood pressure below 50 mmHg. Additional entrainment or pace mapping was performed at the operator’s discretion.

### Conventional point-by-point 3D-mapping

Conventional point-by-point 3D-mapping was performed using an electroanatomical 3D-mapping system (Carto® 3 System, Biosense Webster, Irvine, CA, USA). The detailed approach has been described elsewhere^14^. In brief, similarly to the UHD group, a sequential approach was performed during all procedures. An endocardial voltage map was acquired through a retrograde and/or antegrade transseptal access using a steerable ablation catheter (THERMOCOOL®, D- or F-Type, 2-5-2 mm spacing; Biosense Webster, see Fig. 2B). LAVA, including fractionated and late potentials as well as areas of scar (voltage < 1.5 mV), were marked and later considered for ablation. After that, VT was induced using programmed stimulation. If VT was induced and hemodynamically tolerated, entrainment mapping was performed using the Stimulus-to-QRS delay, the ratio of Stimulus-to-QRS delay to VT cycle length (CL) and the endocardial activation time in comparison to the surface ECG to identify the critical isthmus. Furthermore, areas with middiastolic activation during ongoing VT were considered an ablation target. Pace mapping was performed at the operator’s discretion.

### VT ablation and follow-up

The VT ablation strategy has been described before^10^. In brief, we aimed to achieve the combined procedural endpoint of VT non-inducibility and substrate modification in both groups. Radiofrequency current was used for complete abolition of all abnormal electrograms including fractionated potentials, highly fractionated potentials, late potentials, and fractionated late potentials^10,15,16^. Subsequently, targeted regions were remapped to demonstrate elimination of the respective electrograms. Radiofrequency current was applied with a maximum power of 40 W (upper temperature limit was set to 48 °C) at an irrigation rate of 17–30 mL/min. After discharge, amiodarone was continued at the operator’s discretion. Patients were seen in our outpatient clinic, including device interrogation, after three months and every three to six months subsequently, depending on individual symptoms. The electronic patient charts were reviewed by a senior electrophysiologist for the acquisition of patient characteristics, procedural parameters, and follow-up. In case of missing follow-up visits the patient’s cardiologist and general practitioner were contacted. A recurrence was defined as any sustained or non-sustained VT, documented by device interrogation or ECG. Disease-related death was defined as sudden cardiac death due to ventricular arrhythmia or cardiogenic shock in combination with or caused by ventricular tachycardia.

### Statistical analysis

Descriptive statistics are presented as count and percentage for categorical variables and as mean ± standard deviation for continuous variables if normally distributed (assessed visually using histograms) or median (interquartile range (IQR)) otherwise. Patient and treatment characteristics were compared with Fisher’s exact test or Mann–Whitney U test, respectively. The cumulative probabilities of recurrence or disease-related death over time were estimated and compared between groups using cumulative incidence functions accounting for the competing event non-disease-related death. Estimates for recurrence or disease-related death are presented with 95% confidence intervals (CI). Cumulative incidence curves are truncated at 24 months. A cause-specific Cox proportional hazards model was used to assess the relationship between covariates and time to recurrence or disease-related death. The covariates “mapping technique”, “age (scaled in decades)”, “sex”, “body mass index (BMI)”, “type of cardiomyopathy”, “chronic kidney disease”, “left ventricular ejection fraction (LV-EF)” and “antiarrhythmic drug therapy (AAD)” before ablation were chosen according to recent data on recurrence prediction after VT ablation as well as clinical relevance and included into the model^17^. According to the Grambsch-Thernau test, the proportional hazards assumption is reasonable for all covariates. Two-way interactions between mapping technique and other covariates were found not to be significant and hence eliminated from the model in a backward selection based on the Akaike information criterion. Hazard ratios are presented with 95% confidence intervals as well as Wald *P* values and displayed in a forest plot. The reported *P* values are used as descriptive measures only. *P* values less than 0.05 are regarded statistically significant. Statistical analyses were performed using the statistical software GraphPad Prism 7.0 (GraphPad Software Inc., San Diego, CA, USA) and R version 3.6.3 (R Foundation for Statistical Computing, Vienna, Austria).

### Ethics approval

The study has been approved by the ethics committee of the medical council Hamburg and has therefore been performed in accordance with the ethical standards laid down in the 1964 Declaration of Helsinki and its later amendments.

### Consent to participate

Informed consent was obtained from all individual participants included in the study.

### Consent for publication

Patients signed informed consent regarding publishing their data and photographs.

## Results

### Study population

A total of 122 patients with documented scar-related VT were included into the study. The patients’ baseline parameters did not differ significantly (Table 1).

**Table 1:** Baseline descriptive statistics.

	Total (n = 122)	Ultra-high-density mapping (n = 61)	Conventional 3D-mapping (n = 61)	*P* value
Age, years	64.8 ± 11.9 67.0 (59.0–74.0)	64.8 ± 11.2 64.0 (58.0–73.0)	64.9 ± 12.7 68.0 (59.0–74.0)	0.652
Male sex, n (%)	113 (92.6)	57 (93.4)	56 (91.8)	1.000
BMI, kg/m^2^	28.0 ± 4.3 27.0 (25.0–30.0)	28.3 ± 3.9 28.0 (26.0–30.0)	27.8 ± 4.7 27.0 (25.0–29.0)	0.149
Cardiomyopathy type, n (%)				0.852
Ischemic	76 (62.3)	37 (60.7)	39 (63.9)	
Non-ischemic	46 (37.7)	24 (39.3)	22 (36.1)	
Art. hypertension, n (%)	84 (68.9)	43 (70.5)	41 (67.2)	0.845
Diabetes, n (%)	26 (21.3)	13 (21.3)	13 (21.3)	1.000
Chronic kidney disease, n (%)	53 (43.4)	22 (36.1)	31 (50.8)	0.144
Atrial fibrillation, n (%)	48 (39.3)	26 (42.6)	22 (36.1)	0.578
OAK, n (%)	58 (47.5)	32 (52.5)	26 (42.6)	0.365
LV-EF, %	34.8 ± 13.4 35.0 (23.5–44.8)	35.7 ± 11.6 35.0 (25.0–40.0)	33.8 ± 14.9 35.0 (20.0–45.0)	0.236
Syncope, n (%)	26 (21.3)	13 (21.3)	13 (21.3)	1.000
ICD, n (%)	106 (86.9)	57 (93.4)	49 (80.3)	0.058
AAD before Ablation, n (%)	113 (92.6)	56 (91.8)	57 (93.4)	1.000
Betablockers	88 (72.1)	35 (57.4)	53 (86.9)	0.001
Amiodarone	59 (48.4)	30 (49.2)	29 (47.5)	1.000

Unless noted, values are mean ± standard deviation / median (interquartile range) or n (percent). *P* value < 0.05 is considered significant.

AAD indicates antiarrhythmic drug therapy; Art., arterial; BMI, body mass index; ICD, implantable cardioverter-defibrillator; LV-EF, left ventricular ejection fraction; OAK, oral anticoagulation; 3D, three-dimensional.

### Procedural data

The procedural parameters were similar in both groups (see Table 2A). The median procedure duration was 201.5 (IQR 175.8-240.0) minutes in the UHD group and 180.0 (IQR 145.0-235.0) minutes in the conventional mapping group (*P* = 0.117). The median fluoroscopy duration (19.0 (IQR 15.2-26.0) minutes versus 22.9 (IQR 13.8-31.1) minutes) did not differ between groups (*P* = 0.481). The median radiofrequency duration was 1637 (871-2536) seconds in the UHD group and 1641 (IQR 1096-2333) seconds in the conventional mapping group (*P* = 0.763). 24.6% (15/61; UHD group) versus 27.9% (17/61; conventional mapping group) of patients had a previous VT ablation (*P* = 0.837).

**Table 2:** Procedural parameters and outcome.

	Total (n = 122)	Ultra-high-density mapping (n = 61)	Conventional 3D-mapping (n = 61)	*P* value
*A. Procedural parameters*				
Procedure duration, min	198.0 (160.0–240.0)	201.5 (175.8–240.0)	180.0 (145.0–235.0)	0.117
Fluoroscopy duration, min	20.0 (14.8–29.5)	19.0 (15.2–26.0)	22.9 (13.8–31.1)	0.481
Radio frequency duration, sec	1639 (963–2401)	1637 (871–2536)	1641 (1096–2333)	0.763

	Total (n = 122)	Ultra-high-density mapping (n = 61)	Conventional 3D-mapping (n = 61)	*P* value
*B. Long-term outcome*				
Median survival time free from recurrence or death from any cause, months		15.8 [6.8–24.3]	5.1 [2.5–15.7]	
Cumulative incidence of recurrence or disease-related death at 12 months, %		41.0 [28.5–53.1]	59.0 [45.5–70.3]	
Cumulative incidence of recurrence or disease-related death at 24 months, %		52.4 [36.9–65.7]	69.6 [55.9–79.8]	
AAD at 24 months, n (%)	118 (96.7)	59 (96.7)	59 (96.7)	1.000
Betablockers	110 (90.2)	52 (85.2)	58 (95.1)	0.1258
Amiodarone	65 (53.3)	33 (54.0)	32 (52.5)	1.000
Mexiletine	2 (1.6)	0	2 (3.3)	0.4959
Calcium channel blockers	2 (1.6)	0	2 (3.3)	0.4959

Unless noted, values are median [IQR], cumulative incidence estimates [95% confidence interval] or n (percent). *P* Value < 0.05 is considered significant. AAD indicates antiarrhythmic drug therapy; min, minutes; sec, seconds; 3D, three-dimensional.

A substrate map was created in all patients in both groups. A monomorphic VT was inducible in 82.0% (50/61) of patients in the UHD group (mean CL 350 ± 100 ms) and in 85.2% (52/61, *P* = 0.807) of patients in the conventional mapping group (mean CL 325 ± 110 ms). The mean number of inducible VTs was 2.1 ± 1.5 in the UHD group and 2.0 ± 1.0 in the conventional mapping group (*P* = 1.000). Two patients in the UHD group and no patients in the conventional mapping group presented with ongoing VT at the beginning of the procedure. 44.2% (23/52) of all VT in the UHD group and 48.1% (25/52) in the conventional mapping group were hemodynamically not or only shortly tolerated (*P* = 0.844). Activation mapping and/or entrainment mapping was performed in all patients with hemodynamically tolerated VT (47.5% (29/61) in the UHD group versus 44.3% (27/61) in the conventional mapping group; *P* = 0.856). Furthermore, pace mapping was performed in 50.8% (31/61) in the UHD group and in 90.2% (55/61) in the conventional mapping group (*P* = 0.001).

### Acute procedural outcome

In patients with inducible VT, acute procedural success (non-inducibility of the clinical VT at the end of the procedure) was documented in 82.0% (41/50) in the UHD group versus 92.3% (48/52) in the conventional mapping group (*P* = 0.145).

Periprocedural complications occurred in five patients in the UHD group (two patients with hematoma of the groin not requiring intervention, two with aspiration pneumonia, one with pericardial tamponade) and five patients in the conventional mapping group (one patient with hematoma of the groin not requiring intervention, one with retroperitoneal hematoma, one with aspiration pneumonia, one with right bundle branch block, one with periprocedural stroke).

### Long-term procedural outcome

**Figure 3:**
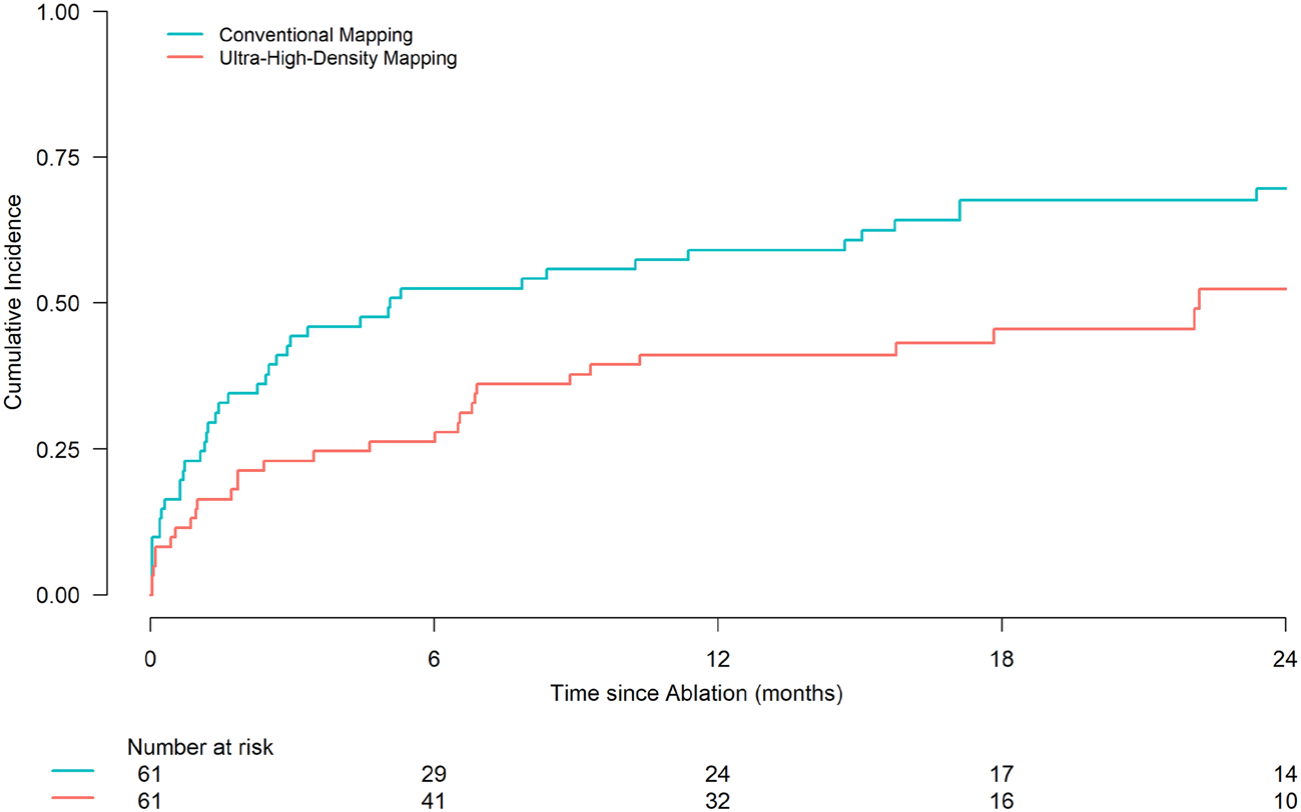
Lower rates of recurrence or disease-related death after catheter ablation of ventricular tachycardia guided by ultra-high-density mapping versus conventional point-by-point 3D-mapping. Cumulative incidence of recurrence or disease-related death in the first 24 months after ablation are displayed. Patients with ablation guided by ultra-high-density mapping had a lower recurrence or disease-related death rate at 12-/24-months of followup (see also Table 2B). Patients with ultra-high-density mapping are depicted in red, patients with conventional point-by-point 3D-mapping in cyan. Steps represent a first recurrence or disease-related death. Non-disease-related death (not displayed) was considered as competing risk.

**Figure 4:**
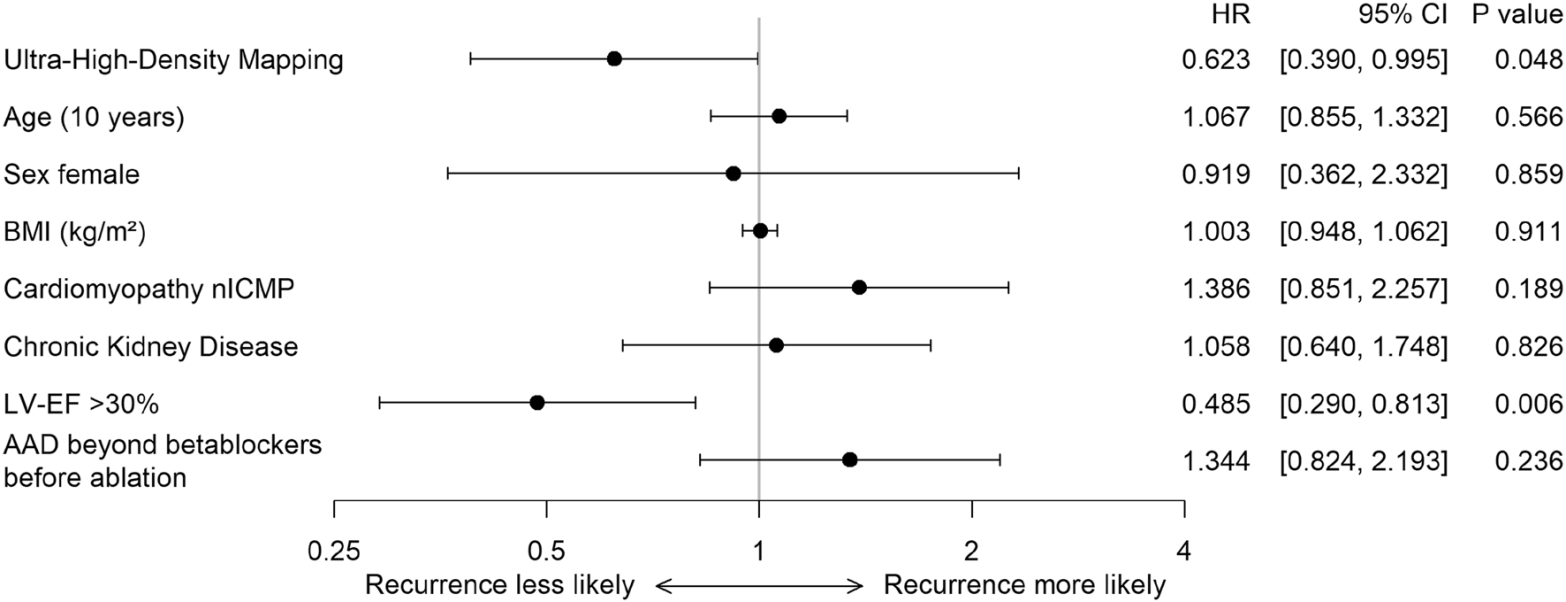
Ultra-high-density mapping and left ventricular ejection fraction > 30% are independently associated with improved long-term outcome. A Forest plot of a multivariable cause-specific Cox proportional hazards model for time to recurrence or disease-related death is displayed. Values on the right side of the line represent a higher likelihood and values on the left a lower likelihood of a first recurrence or disease-related death. Results are presented as hazard ratio with 95% confidence interval. *P* < 0.05 is considered significant. The parameter “age” represents the effect of a 10-year increase in patient age on the likelihood of recurrence or disease-related death. The model adjusts for mapping technique, age, sex, body mass index (BMI), type of cardiomyopathy, chronic kidney disease, left ventricular ejection fraction (LV-EF) and antiarrhythmic drug therapy (AAD) before ablation. AAD indicates antiarrhythmic drugs; BMI, body mass index; CI, confidence interval; HR, hazard ratio; LV-EF, left ventricular ejection fraction; nICMP, non-ischemic cardiomyopathy.

The median follow-up time was 21.1 months (95% CI [17.8-∞]) in the UHD group and 56.2 months (95% CI [38.1-∞]) in the conventional mapping group (calculated by reverse Kaplan–Meier). The follow-up duration differed between groups because the ablation procedures of the conventional mapping group were performed prior to the procedures of the UHD group (see methods section). Sensitivity analyses censoring events occurring after the maximum follow-up time observed in the UHD group were performed (see Supplemental Fig. 1). The estimated 12-months cumulative incidence of recurrence or disease-related death was 41.0% (95% CI [28.5–53.1]; recurrence: n = 21; disease-related death n = 4) in the UHD group and 59.0% (95% CI [45.5–70.3]; recurrence: n = 26; disease-related death n = 10) in the conventional mapping group (see Table 2B). The 24-months estimate of the cumulative incidence was 52.4% (95% CI [36.9–65.7]; recurrence: n = 25 disease-related death: n = 4) in the UHD group and 69.6% (95% CI [55.9–79.8]; recurrence: n = 31; disease-related death n = 11) in the conventional mapping group (see Fig. 3). VT recurrence or death from any cause occurred after a median failure-free survival time of 15.8 months (95% CI [6.8–24.3]) after ablation in the UHD group and 5.1 months (95% CI [2.5–15.7]) in the conventional mapping group). A cause-specific Cox proportional hazards model showed that UHD mapping had an independent influence on the time to first recurrence or disease-related death with a HR of 0.623 (95% CI [0.390–0.995]; *P* = 0.048). Furthermore, an LV-EF > 30% in comparison to an LV-EF ≤ 30% demonstrated an independent effect: HR of 0.485 (95% CI [0.290–0.813]; *P* = 0.006; see Fig. 4). During follow-up, 13 patients in the UHD group and 14 patients in the conventional mapping group died. Death was classified as disease related in 6 patients of the UHD group and in 11 patients of the conventional mapping group.

The use of AAD at last follow-up was similar in both groups (see Table 2B). Cumulative incidence curves for recurrence or disease-related death stratified by type of cardiomyopathy and LV-EF are shown in Supplemental Figs. 2 and 3.

## Discussion

The main findings of the present study are:

1. Catheter ablation guided by UHD mapping is associated with fewer recurrences or disease-related deaths during the first 24 months after ablation in comparison to procedures guided by conventional point-by-point 3D-mapping using a 3.5 mm single tip catheter.
2. The procedural duration and the radiation exposure did not differ between both mapping methods.
3. Both methods did not differ with respect to periprocedural complications.

Substrate-based VT ablation approaches are dependent on correct delineation of myocardial scar and abnormal electrograms. Previous studies have shown an advantage of complete abolition of abnormal electrograms on the outcome of VT ablation^6^. UHD mapping creates a high spatial mapping density and may thus facilitate the depiction of those electrograms^18,19^. However, the impact of UHD mapping on ablation outcome remains under discussion. In the present study, patients who underwent ablation guided by UHD mapping had fewer recurrences or disease-related deaths during follow-up than patients with conventional mapping. Studies comparing novel mapping techniques with conventional point-by-point mapping during VT ablation are sparse and partly contradictory. The only randomized study (n = 20 patients) showed no difference in VT recurrences after 12 months post ablation^20^. Similarly, Cano et al. did not see a benefit in long-term outcome comparing multielectrode mapping and conventional mapping in an observational study analysing 81 patients^21^. In contrast, another observational study including 125 patients was able to identify mapping with multipolar catheters as a predictor for reduced recurrences besides image integration and success of abolition of all LAVA^22^.

The duration of radiofrequency energy application was similar in both of our patient groups and a larger ablated area is unlikely to be responsible for our observations. This finding goes in line with reports by Tschabrunn et al., showing that the ablation target area during UHD mapping is not bigger, but might even appear smaller. Hypothetically this is explained by remaining channels of healthy myocardium in heterogeneous tissue which are identified during UHD mapping and can be missed with larger sized electrode catheters^19^. An improved discrimination of far- and nearfield signal leads additionally to better discrimination during electrogram analysis as shown by Berte et al.^23^.

The aforementioned, rather unspecific approach of ablating all LAVA might sometimes be a necessary evil to prevent relevant areas from being missed during ablation, but it can come at the cost of excessive ablation and procedure times^24^. We showed in a previous study, that highly fractionated signals representing slowly conducting channels, detected by UHD mapping were often present at the critical VT isthmus site and represent a more specific target. Those signals were often confined to a small space and a high mapping density might be beneficial for detection. As 3.5 mm single tip electrode catheters were used for mapping in the conventional mapping group, it is possible that signals were sometimes missed due to the lower resolution, which might be one reason for a worse ablation outcome^23^. Still in general, detection of highly fractionated isthmus signals should be possible using modern single tip catheters, especially if they contain additional micro electrodes.

Another reason leading to better long-term outcome in patients treated using UHD mapping might be the shorter mapping time for creation of activation maps, facilitating the identification of critical parts of the reentry mechanism^25^. Mapping during ongoing VT was often not tolerated due to hemodynamic instability. Faster mapping using the mini-basket catheter might facilitate the completion of an activation map with the benefit of specific ablation at the critical isthmus site^11,21^.

Better LV function was another factor associated with reduced recurrence rates. This observation has been described before^17^. Besides better mapping conditions in patients with better hemodynamic status, the main reason for this finding might be less advanced underlying heart disease.

During the initial introduction of multipolar catheters, there were concerns about a higher thrombogenicity due to the more complex structure of the catheter. We aimed at an ACT > 300 s during mapping and ablation for all procedures in the UHD group. As the procedures in the conventional mapping group were performed before introduction of UHD mapping, the ACT goal was still > 250 s for those patients. Both groups had low complication rates, especially low rates of stroke (n = 1, conventional mapping group) and pericardial tamponade (n = 1, UHD group). Our data stand in line with several studies, showing good procedural safety for both mapping approaches^26,27^.

### Limitations

While our study includes a relatively large number of patients in comparison to other studies available in the literature on this subject, the number of patients at risk might be too small for drawing conclusions for the follow-up beyond year two and later. Since the present study has a retrospective design and should thus be considered hypothesis generating, prospective, large, randomised studies are needed to confirm the benefits of UHD mapping guided VT ablation. Due to the retrospective design, inclusion bias can't be fully excluded and might have had an influence on our results. Although all procedures were conducted by operators with several years of experience in interventional electrophysiology, a learning curve effect is possible and might have influenced the outcome of the UHD mapping group which was treated later than the conventional mapping group. Even if AAD intake was reported during follow-up appointments. the exact duration of AAD intake and the patients’ compliance regarding medication is not known and might have influenced recurrence rates. Although the energy levels chosen for ablation were similar in both groups, the different models of ablation catheters used in the UHD and the conventional mapping group might have had an influence on the lesion quality and thus also on the ablation outcome.

## Conclusion

VT ablation guided by UHD mapping was independently associated with an improved 24-months outcome in comparison to procedures with conventional point-by-point 3D-mapping. Both mapping techniques were safe, had similar procedure times, and similar radiation exposure.

## Supplementary Information

Supplementary Information.

## Data Availability

Data are available upon reasonable request.
