# Supplementary material for: The impact of ultra-high-density mapping on long-term outcome after catheter ablation of ventricular tachycardia
Source: Sci Rep. 2022 Jun 1;12:9139. doi: 10.1038/s41598-022-12918-7 (PMC9160260; doi:10.1038/s41598-022-12918-7)
Supplement: Supplementary file 1 — Supplementary Information. [file 41598_2022_12918_MOESM1_ESM.docx]

**Supplemental Materials**

**Supplemental Fig. 1 Sensitivity Analysis**


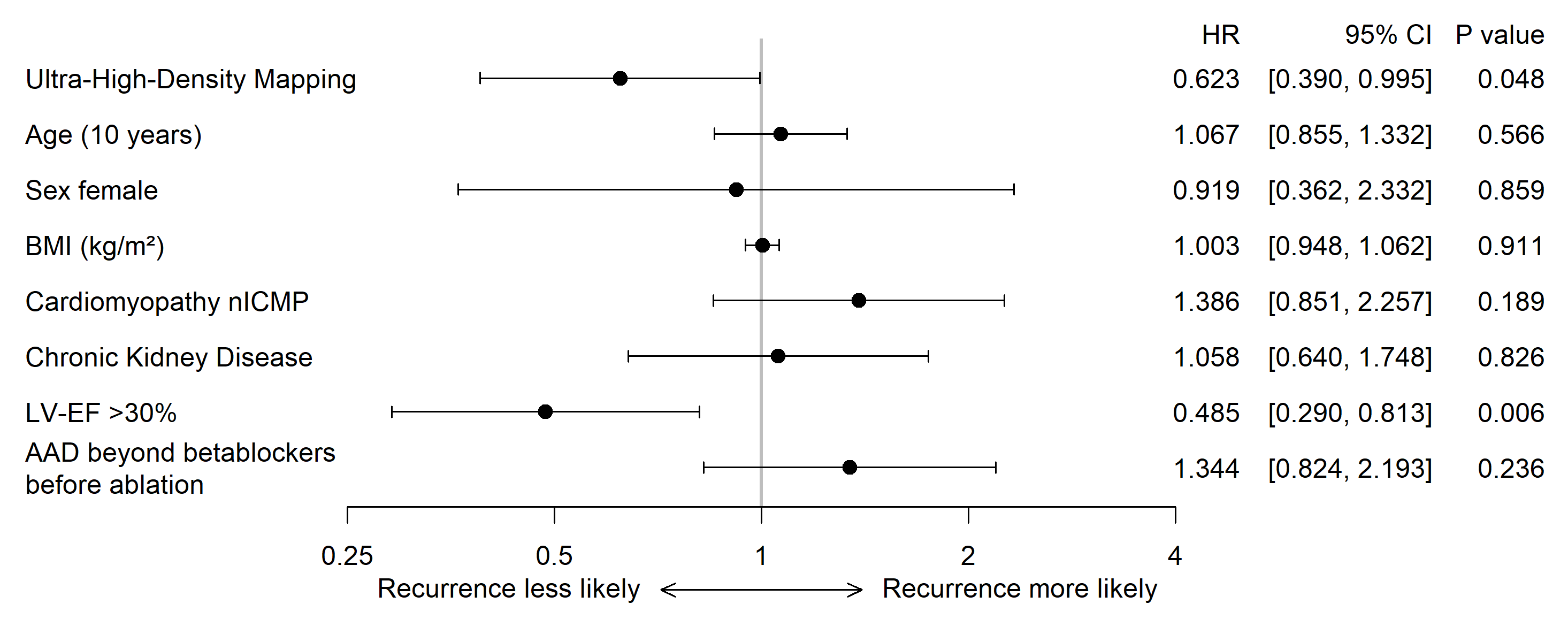


This Forest Plot is depicting a multivariable cause-specific Cox proportional hazards model for time to recurrence or disease-related death. It is resulting from a sensitivity analysis censoring events occurring after the maximum follow-up time observed in the Ultra-High-Density Mapping group (compare Figure 4 of the manuscript). Values on the right side of the line represent a higher likelihood and values on the left a lower likelihood of a first recurrence or disease-related death. Results are presented as hazard ratio with 95% confidence interval. *P*<0.05 *is* considered significant. Ultra-High-Density Mapping and Left ventricular ejection fraction >30% were independently associated with lower event rates. The variable age represents the effect of a 10-year increase in patient age on the likelihood of recurrence or disease-related death. The model adjusts for mapping technique, age, sex, body mass index (BMI), type of cardiomyopathy, chronic kidney disease, left ventricular ejection fraction (LV-EF) and antiarrhythmic drug therapy (AAD) before ablation.

AAD indicates antiarrhythmic drugs; BMI, body mass index; CI, confidence interval; HR, hazard ratio; LV-EF, left ventricular ejection fraction; nICMP, non-ischemic cardiomyopathy.

**Supplemental Fig. 2 Differences in Long-term Outcome of Patients with Ischemic or non-Ischemic Cardiomyopathy**


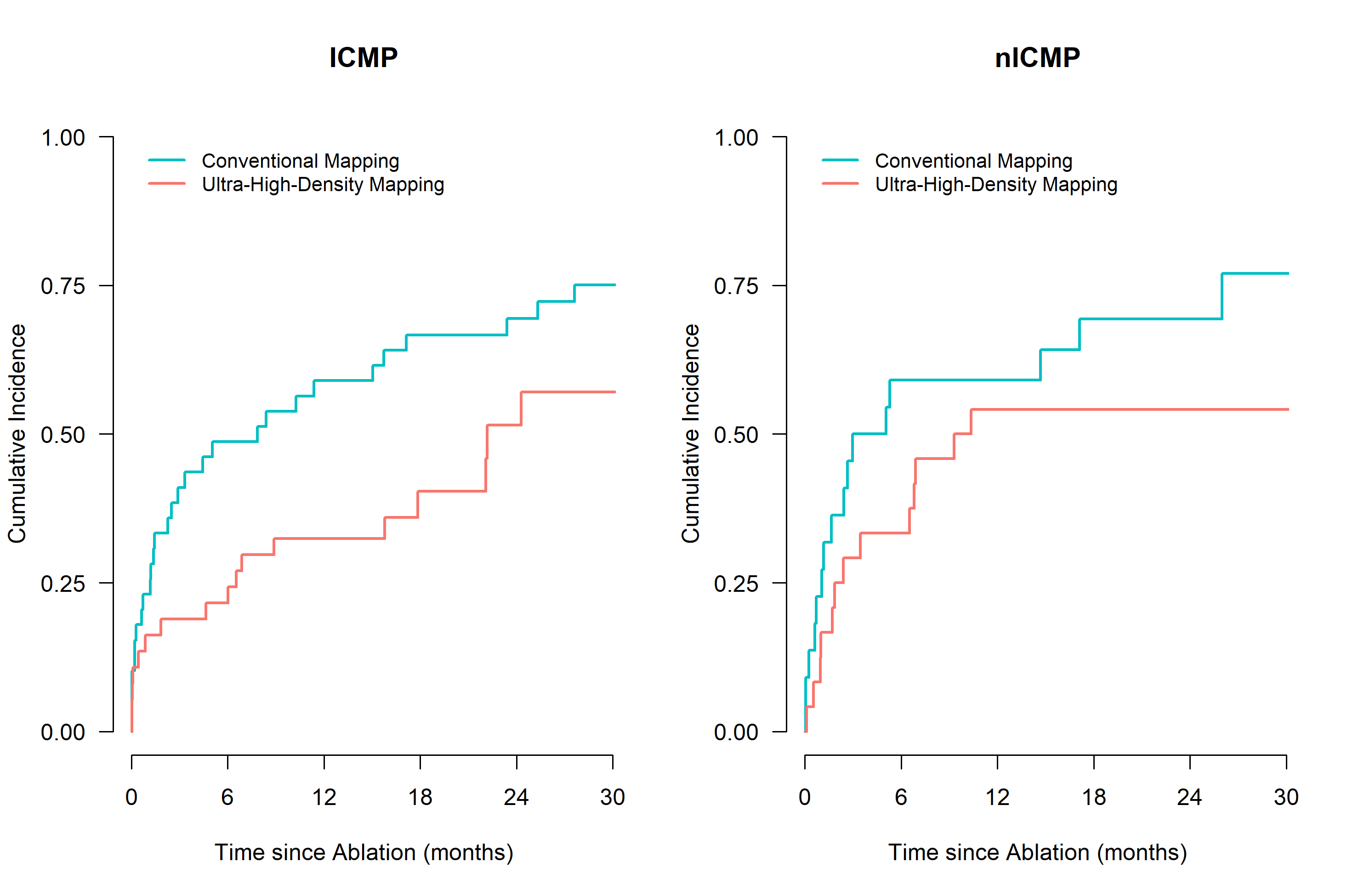


Curves of cumulative incidence of first recurrence or disease-related death displayed up to 30 months after ablation, comparing patients with ultra-high-density mapping (red) and conventional point-by-point mapping (turquoise). Left side shows patients with ischemic cardiomyopathy, right side shows patients with non-ischemic cardiomyopathy. Steps represent a first recurrence or disease-related death. Non-disease-related death (not displayed) was considered as competing risk.

Abbreviations: ICMP, ischemic cardiomyopathy; nICMP, non-ischemic cardiomyopathy.

**Supplemental Fig. 3 Differences in Long-term Outcome of Patients with Left Ventricular Ejection Fraction ≤/> 30%**

**
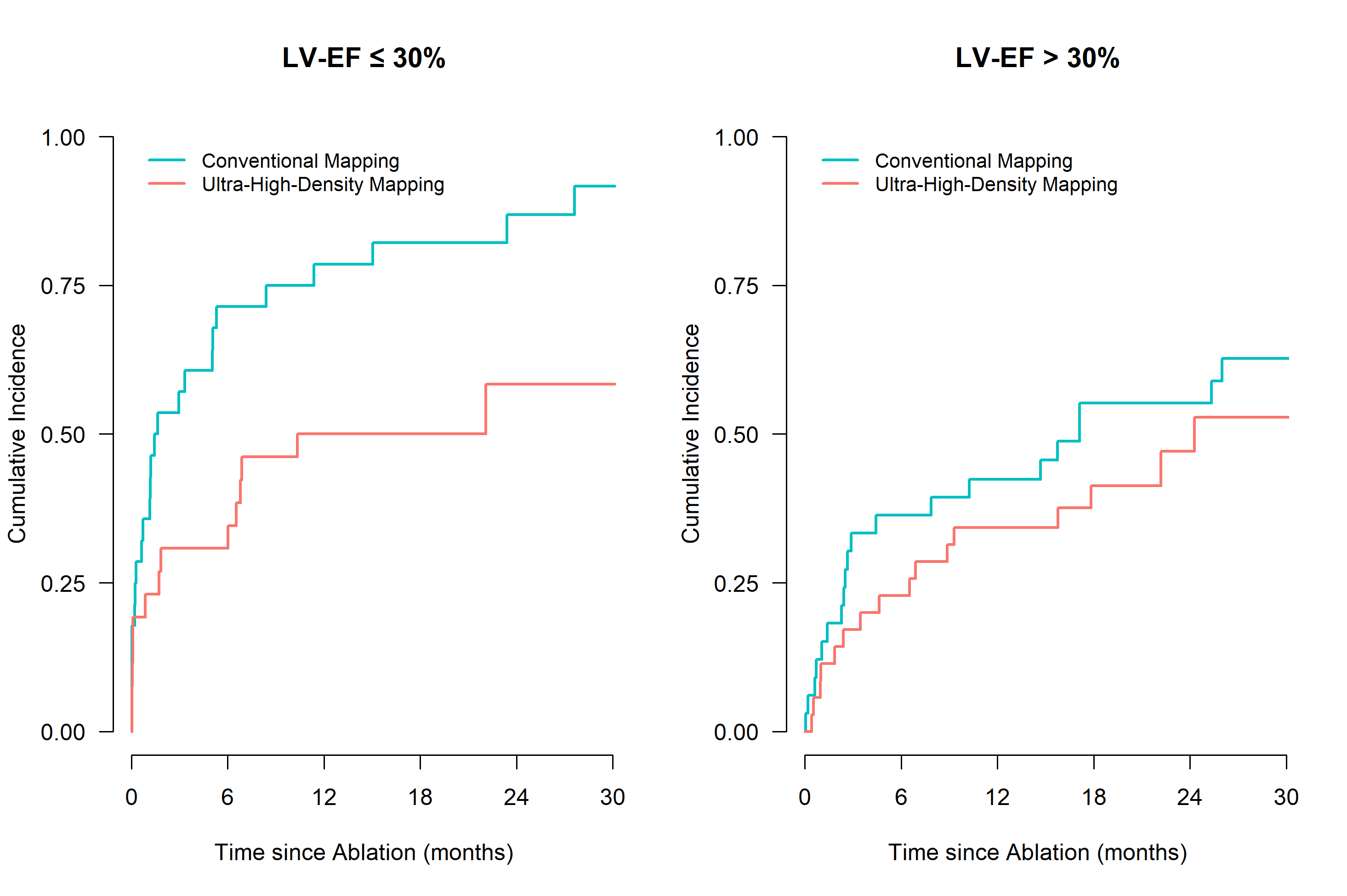
**

Curves of cumulative incidence of first recurrence or disease-related death displayed up to 30 months after ablation, comparing patients with ultra-high-density mapping (red) and conventional point-by-point mapping (turquoise). Left side shows patients with left ventricular ejection fraction ≤30%, right side shows patients with left ventricular ejection fraction >30%. Steps represent a first recurrence or disease-related death. Non-disease-related death (not displayed) was considered as competing risk.

Abbreviations: LV-EF, left ventricular ejection fraction.
